# Strangulated Hernia, New Treatment

**Published:** 1875-08

**Authors:** Alexander Hadden

**Affiliations:** New York


STRANGULATED HERNIA REDUCED BY TAXIS THROUGH COLON.—*By Alexander Hadden, M.D., New York.*—I then suggested that before we resorted to the knife, an effort might be made to reduce the hernia by passing the hand through the rectum into the colon, and endeavoring through its walls to produce traction on the involved intestine sufficient to disengage it. This was approved of, and I accordingly did so, and succeeded.

The steps of the operation were as follows: the patient being fully under the influence of chloroform, was placed on her chest and knees, and supported in that position; I next introduced my fingers into the anus, and passed my hand by gentle pressure up into the colon. I had some difficulty in following the intestine over the promontory of the sacrum, but when my hand had passed this point it was very free. I could feel the engorged intestine plainly, and could make traction on it with my fingers, and did carefully, fearing that I might rend it. My manipulation consisted chiefly in gently rubbing my finger along the intestine from the inner surface of the ring, and using external taxis with my other hand at the same time. The desired result was accomplished in about ten or fifteen minutes, with comparative ease.—*Medical Record.*

